# Neoplastic cell enrichment of tumor tissues using coring and laser microdissection for proteomic and genomic analyses of pancreatic ductal adenocarcinoma

**DOI:** 10.1186/s12014-022-09373-x

**Published:** 2022-10-20

**Authors:** Qing Kay Li, Yingwei Hu, Lijun Chen, Michael Schnaubelt, Daniel Cui Zhou, Yize Li, Rita Jui-Hsien Lu, Mathangi Thiagarajan, Galen Hostetter, Chelsea J. Newton, Scott D. Jewell, Gil Omenn, Ana I. Robles, Mehdi Mesri, Oliver F. Bathe, Bing Zhang, Li Ding, Ralph H. Hruban, Daniel W. Chan, Hui Zhang

**Affiliations:** 1 Department of Pathology, the Johns Hopkins University, 400 N Broadway, Smith Bldg Rm 4011, Baltimore, MD 21231 USA; 2 Department of Oncology, Sidney Kimmel Cancer Center, Johns Hopkins Medical Institutions, 600 N. Wolfe Street, Baltimore, MD USA; 3 Department of Oncology, Washington University at Saint Louis, St Louis, MO USA; 4 Leidos Biomedical Research Inc, Frederick National Laboratory for Cancer Research, Frederick, MD USA; 5 Van Andel Research Institute, Grand Rapids, MI USA; 6 Department of Computational Medicine & Bioinformatics, University of Michigan, Ann Arbor, MI USA; 7 Office of Cancer Clinical Proteomics Research, National Cancer Institute, Rockville, MD USA; 8 Department of Surgery and Oncology, Cumming School of Medicine, University of Calgary, Calgary, AB Canada; 9 Lester and Sue Smith Breast Center, Department of Molecular and Human Genetics, Baylor College of Medicine, Houston, TX USA

**Keywords:** Liquid chromatography–mass spectrometry, TMT-labeling, Tissue sampling techniques, Bulk tissue, Tissue coring, Laser microdissection (LMD), Clinical Proteomic Tumor Analysis Consortium (CPTAC)

## Abstract

**Background:**

The identification of differentially expressed tumor-associated proteins and genomic alterations driving neoplasia is critical in the development of clinical assays to detect cancers and forms the foundation for understanding cancer biology. One of the challenges in the analysis of pancreatic ductal adenocarcinoma (PDAC) is the low neoplastic cellularity and heterogeneous composition of bulk tumors. To enrich neoplastic cells from bulk tumor tissue, coring, and laser microdissection (LMD) sampling techniques have been employed. In this study, we assessed the protein and *KRAS* mutation changes associated with samples obtained by these enrichment techniques and evaluated the fraction of neoplastic cells in PDAC for proteomic and genomic analyses.

**Methods:**

Three fresh frozen PDAC tumors and their tumor-matched normal adjacent tissues (NATs) were obtained from three sampling techniques using bulk, coring, and LMD; and analyzed by TMT-based quantitative proteomics. The protein profiles and characterizations of differentially expressed proteins in three sampling groups were determined. These three PDACs and samples of five additional PDACs obtained by the same three sampling techniques were also subjected to genomic analysis to characterize *KRAS* mutations.

**Results:**

The neoplastic cellularity of eight PDACs ranged from less than 10% to over 80% based on morphological review. Distinctive proteomic patterns and abundances of certain tumor-associated proteins were revealed when comparing the tumors and NATs by different sampling techniques. Coring and bulk tissues had comparable proteome profiles, while LMD samples had the most distinct proteome composition compared to bulk tissues. Further genomic analysis of bulk, cored, or LMD samples demonstrated that *KRAS* mutations were significantly enriched in LMD samples while coring was less effective in enriching for *KRAS* mutations when bulk tissues contained a relatively low neoplastic cellularity.

**Conclusions:**

In addition to bulk tissues, samples from LMD and coring techniques can be used for proteogenomic studies. The greatest enrichment of neoplastic cellularity is obtained with the LMD technique.

**Supplementary Information:**

The online version contains supplementary material available at 10.1186/s12014-022-09373-x.

## Introduction

Pancreatic ductal adenocarcinoma (PDAC) accounts for > 90% of malignant tumors of the pancreas [1–3]. Clinically, the majority of PDAC present at an advanced-stage with unresectable cancer at the time of diagnosis [2–4]. Despite the rapid development of targeted- and immuno-therapies for cancers, the outcome of PDAC is still dismal with a 5-yr survival rate of 11% [2–4]. It is well-known that PDAC is driven by the accumulation of genomic aberrations, and that these, in turn, drive the phenotypical and molecular transformation from normal pancreatic epithelial cells into a non-invasive neoplastic precursor lesion and eventually to an infiltrating malignant cancer [5–9]. Recent large-scale proteogenomic studies such as the Clinical Proteomic Tumor Analysis Consortium (CPTAC) PDAC study have significantly advanced our knowledge of the molecular biology of PDAC [10–16]. Somatic mutations in the *KRAS* gene have been identified in > 90% of PDACs [17–20]. Other molecular events have also been identified as drivers in the development and progression of PDAC, including the loss of function of tumor suppressor genes *TP53*, *p16*/*CDKN2A* and *SMAD4,* activation of oncogenic Her2/neu, and germline mutations of *BRCA*1/2, *PALB2*, *ATM*, *MLH1* and others [11–13]. In conjunction with genomic findings, proteomic studies have identified up- and down-regulated proteins and signaling pathways in PDAC [14–16]. The recent comprehensive proteomic analysis of 135 PDACs has identified a number of protein changes in tumors compared to NATs and normal ductal epithelium [16]. Proteins such as HK2, LOXL2, COL12A1, C19orf33, TSPAN1 and MDK have been considered as diagnostic or prognostic markers, as well as potential therapeutic targets [16].

As these multi-omics studies, in particular proteomic analyses, have potential clinical applications, it is clear that identification of differentially expressed tumor-associated proteins is a critical step for the study of cancer biology, pathophysiologic perturbations, and the development of potential clinical assays. It is well established, however, that this process toward clinical application is challenging, since cellular heterogeneity, the mixture of neoplastic and non-neoplastic cells, can obscure neoplasm-specific protein expression patterns [13, 16, 21–24]. The analysis of bulk tumor tissue is a conventional approach to profiling tumor-associated proteins; however, bulk tumor, since it relies on gross tumor identification, can contain substantial amounts of non-neoplastic tissues. This is particularly a problem in PADC, where the majority of tumors have < 20% neoplastic cellularity, and only some, often unusual, tumors have reasonably high neoplastic cellularity [16, 19, 24]. Bulk tumor tissue in the pancreas typically contains normal pancreas, mucin, collagen, fibroblasts, vascular endothelial cells, and inflammatory cells. Thus, enrichment of neoplastic cells is an important step in the proteomic analysis of tumors, particularly PDAC.

To address the issue of low neoplastic cellularity, several micro-dissection techniques, such as laser microdissection (LMD) and coring of tumor tissue, have been used to enrich samples for neoplastic cells [25–30]. In the LMD approach, microscope sections are examined and areas selected and excised using a laser. This technique can be used to isolate a relatively pure cellular population for further multi-omics analysis. However, it is time-consuming and the yield of recovered tissue material is low. In addition, the laser can heat the tissues to be studied, and this heat can cause artifacts and degradation of nucleic acids. In the coring technique, aspiration needles of different gauges are used to punch the selected targeted areas from larger blocks of tissue [30]. Similar to LMD, it can provide a relatively pure cellular component from the top layer of tissue blocks. However, the cellular components beneath the top layer of cells in the cored tissues are difficult to determine as tissues are almost never perfectly oriented vertically. Although these approaches are routinely applied to genetic analyses, they are often overlooked in proteomic analyses.

In this study, we compared different sampling techniques, including bulk tumor tissue, LMD, and coring, for the proteomic and genomic analyses of PDAC. The goal was to evaluate the impact of the different sampling techniques on the observed proteomic and genomic data, and to address the potential utility of LMD and coring techniques in the enrichment of neoplastic cells.

## Methods

### Materials and reagents

BCA protein assay kit (Pierce), urea, tris (2-carboxyethyl) phosphine (TCEP), tandem mass tag (TMT) reagents and dithiothreitol (DTT) were purchased from Thermo Fisher Scientific (Waltham, MA). Sequencing-grade trypsin was purchased from Promega (Madison, WI). Lys-C was purchased from Wako Chemicals (Richmond, VA). C18 SPE columns were purchased from Waters (Milford, MA). All other reagents, including iodoacetamide (IAA), formic acid (FA), and anhydrous acetonitrile (ACN), were purchased from Sigma-Aldrich (St. Louis, MO).

### Samples collection and process

Fresh-frozen blocks of primary PDACs from treatment naïve patients and tumor-matched NATs were prospectively collected from surgically resected specimens according to the CPTAC guidelines [16]. All study cases had no prior history of other malignancies, and the patients had not received systemic chemotherapy, or immune-related therapy. The clinical information and the neoplastic cellularity of study cases were determined by histology review and summarized in Additional file 1: Table S1. Informed consent was obtained and reviewed by Institutional Review Boards at tissue collection sites.

The diagnoses were confirmed by re-reviewing digital images of H&E stained slides by board-certified pathologists. The tumor area and tumor-matched normal adjacent tissues (NATs) were marked on microscope slides and matched to corresponding tissue blocks. Tissue blocks were sampled using three techniques, including the bulk sampling of the entire section of the block, LMD for neoplastic cells, and coring of neoplastic areas using a 3 mm diameter biopsy needle. NATs contained acinar cells and ductal epithelium, and scattered stromal and inflammatory cells, but did not contain neoplastic cells. All samples were cryo-pulverized, aliquoted and stored for subsequent proteomic and genomic analyses.

Proteomic analyses were performed in the Mass Spectrometry Core Facility at the Johns Hopkins Biomarker Discovery and Translation Center. DNA sequencing was performed at the Broad Institute, and genomic analyses were performed in the Oncology Center at Washington University.

### Protein extraction and tryptic digestion for proteomics

Protein extraction and digestion were performed as previously described [16]. Briefly, each sample was lysed in lysis buffer containing 8 M urea, 75 mM NaCl, 50 mM Tris (pH 8.0), 1 mM EDTA, 2 μg/mL aprotinin, 10 μg/mL leupeptin, 1 mM PMSF, 10 mM NaF, phosphatase inhibitor cocktail 2 and 3 [1:100 dilution], and 20 μM PUGNAc. The protein concentration in the supernatant was measured by BCA assay. Proteins were reduced and alkylated with DTT (5 mM, 37 °C, 1 h) and IAA (10 mM, room temperature (RT) for 45 min in the dark). The reduced proteins were diluted 1:4 with 50 mM Tris–HCl (pH 8.0) and incubated with Lys-C followed by trypsin digestion with the enzyme-to-substrate ratio of 1:50 overnight at RT. The digestion was quenched by adjusting pH to < 3 with 50% of formic acid (FA). The peptides were desalted on reversed-phase C18 SPE columns and dried using Speed-Vac.

### Tandem Mass Tag (TMT) labeling and peptide fractionation

Dried peptide samples were dissolved in 50 mM HEPES. 50 ul of each sample from three sampling techniques, including 100 μg of proteins from bulk samples, 30 μg of proteins from coring samples, and 6 μg of proteins from LMD samples, were labeled with 10-plex TMT reagents as described previously [16]. Briefly, TMT reagents were added to each sample, and the mixtures were then incubated at RT for 1 h, and quenched with 5% hydroxylamine at RT for 15 min. Labeled peptides in each TMT set were desalted on reversed-phase C18 SPE columns, dried using Speed-Vac, and dissolved in 900 μL of buffer A (5 mM ammonium formate in 2% ACN). Samples from each TMT set were fractionated by the basic reversed-phase liquid chromatography (bRPLC) with a 4.6 mm × 250 mm Zorbax Extend-C18 analytical column (3.5 μm beads, Agilent) lined up with an Agilent 1220 Series HPLC. A pooled sample from all tumors and NATs was also included in each TMT set as reference.

Peptides were separated using a non-linear gradient with buffer B (5 mM ammonium formate in 90% ACN) as follows: 0% buffer B for 7 min, 0–16% buffer B for 6 min, 16–40% buffer B for 60 min, 40–44% buffer B for 4 min, 44–60% buffer B for 5 min, and holding at 60% buffer B for 14 min. Fractions were concatenated into 24 fractions as described previously [16, 31]. Samples were resuspended in 3% ACN (0.1% FA) prior to ESI-LC–MS/MS analysis.

### ESI-LC–MS/MS for global proteome data-dependent analysis (DDA)

The TMT-labeled fractions were analyzed using Orbitrap Fusion Lumos mass spectrometer (Thermo Scientific). Approximately 0.8 μg of peptides were separated on an in-house packed 28 cm × 75 mm diameter C18 column (1.9 mm Reprosil-Pur C18-AQ beads (Dr. Maisch GmbH); Picofrit 10 mm opening (New Objective)) lined up with an Easy nLC 1200 UHPLC system (Thermo Scientific). The column was heated to 50 °C using a column heater (Phoenix-ST). The flow rate was set at 200 µl/min. Buffer A [3% ACN (0.1% FA)] and buffer B [90% ACN (0.1% FA)] were used. The peptides were separated with a 6–30% buffer B gradient in 84 min, eluted from the column and nanosprayed directly into the mass spectrometer in a data-dependent mode.

Parameters for global proteomic samples were set as follows: MS1 resolution–60,000, mass range–350 to 1800 m/z, RF Lens–30%, AGC Target–4.0e5, Max injection time–50 ms, charge state include–2–6, dynamic exclusion–45 s. The cycle time was set to 2 s, and within this 2 s the most abundant ions per scan were selected for MS/MS in the orbitrap. MS2 resolution–50,000, high-energy collision dissociation activation energy–37, isolation width (m/z)–0.7, AGC Target–2.0e5, max injection time–105 ms.

### Proteomics data processing

Data were searched for peptides and proteins against a human RefSeq protein fasta database using the MS-GF + search engine [32] and MS-PyCloud pipeline [33].

MS/MS spectra were searched using a precursor-ion mass tolerance of 10 ppm. The cysteine carbamidomethylation (+ 57.0215), lysine and peptide N-terminal TMT labeling (+ 229.1629), were specified as fixed modifications. The methionine oxidation (+ 15.9949) was specified as variable modifications. The search was restricted to tryptic peptides, allowing up to two missed cleavage sites. All the other parameters were set as default.

Quantification was based on a similar Unique + Razor peptide approach as described in our previous studies [33–35]. The search results were then filtered by controlling the final protein-level FDR to < 1%. PSMs from all TMT sets were utilized when assigning peptides to protein groups. TMT corrections were applied for the accurate PSM-level quantification. PSMs that passed all filtering criteria were then rolled up to log2 ratio- and abundance-level expression matrices and all samples were then median normalized.

### Comparison of altered proteins in different sampling groups

Proteomic data generated in tumor and NAT samples from three sampling methods (bulk, coring, and LMD) were analyzed by OmicsOne [36]. The fold change of the log2 value of absolute abundances were compared between samples. Due to the limited number of samples, proteins with fold changes ≥ 2.0 were considered as altered proteins. Significantly up- and down-regulated proteins were determined if the fold change ≥ 2.0 and adjusted *p* values < 0.05 via Benjamini–Hochberg approach. The principal component analysis (PCA) was also utilized to evaluate the performance of three sampling methods for differentiating between tumors and NATs. Missing values were not used in the PCA analysis.

### DNA extraction and genomic analysis

DNA was isolated and subjected to Whole Exome Sequencing (WES) as previously described [16]. Eight cases of tissues prepared by bulk, cored, and LMD PDAC underwent WES. Somatic mutations were called by the Somaticwrapper pipeline v1.6 (https://github.com/ding-lab/somaticwrapper), which included four different callers, i.e., Strelka v.2, MUTECT v1.7, VarScan v.2.3.8, and Pindel v.0.2.5from WES. Exonic SNVs was called by any two callers among MUTECT v1.7, VarScan v.2.3.8, and Strelka v.2; and indels was called by any two callers among VarScan v.2.3.8, Strelka v.2, and Pindel v.0.2.5. 14X and 8X coverage cutoff were applied for merged SNVs and indels in tumor and NAT, respectively. SNVs and indels were filtered by a minimal variant allele frequency (VAF) of 0.05 in tumors and a maximal VAF of 0.02 in NAT samples. Any SNV within 10 bp of an indel in the same tumor sample was filtered. The percent of VAFs of *KRAS* mutation were calculated and compared among bulk, LMD and coring WES.

## Results

### Morphological features of analyzed tumors and normal adjacent tissues

**Fig. 1:**
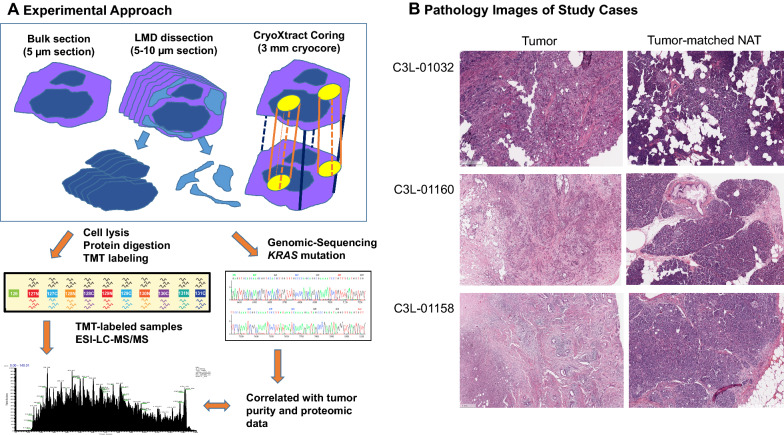
Schematic diagram of the workflow. **A** Three cases of treatment naïve PDAC and tumor-matched NATs were prospectively collected. The tumor area and tumor-matched NAT were marked and matched to corresponding tissue blocks. All tissue blocks were sampled using three techniques, including bulk sampling the tumor (entire section of the block), laser microdissection (LMD) of selected area, and coring of the selected area. **B** Morphological features of study cases. C3L-01032 demonstrated over 80% of tumor cellularity with a minimal amount of desmoplastic stroma. The case C3L-01158 revealed less than 10% of tumor cellularity. In the case C3L-01160, the tumor demonstrated an approximate 40% of tumor cellularity. In addition, the NAT samples also represented a wide range of pancreatic tissue, including predominately acinar cells, benign ductal structures (i.e. C3L-01032) and a low-grade pancreatic intraepithelial neoplasia (i.e. C3L-01160)

In the proteomic analysis, a total of six tissue blocks (three pairs of PDAC tissues and tumor-matched NATs) were sampled using three techniques, including bulk, LMD, and coring. Overall, tumor and NAT tissues were analyzed using the workflow described in Fig. 1A: (1) identifying representative areas of ductal carcinoma in tumor blocks and normal tissues in NATs blocks on the H&E stained slides, and matching targeted area to the tissue blocks, (2) collecting three types of samples from the same tissue block using three approaches (bulk sectioning, LMD dissecting, and coring the targeted area), (3) characterizing three types of samples using proteomic and genomic analyses.

The pathological features of the cancers represented a spectrum of commonly seen morphology in PDACs (Fig. 1B). Based on the histomorphological review of tumor images, C3L-01032 had over 80% neoplastic cellularity with a minimal amount of desmoplastic stroma, C3L-01160 had approximately 40% neoplastic cellularity with abundant desmoplastic stroma and scattered inflammatory cells, and C3L-01158 had less than 10% neoplastic cellularity with significant and dense desmoplastic stroma and foci of tumor necrosis. In the five additional tumors used for genomic analysis, their neoplastic cellularity ranged from 10 to 30% (Additional file 1: Table S1). Finally, the NAT samples represented a wide range of pancreatic tissue, including predominately acinar cells, benign ductal structures (i.e. C3L-01032) and pancreatic intraepithelial neoplasia (i.e. C3L-01160).

### Proteomic analysis in bulk, LMD, and coring tissue samples

**Fig. 2:**
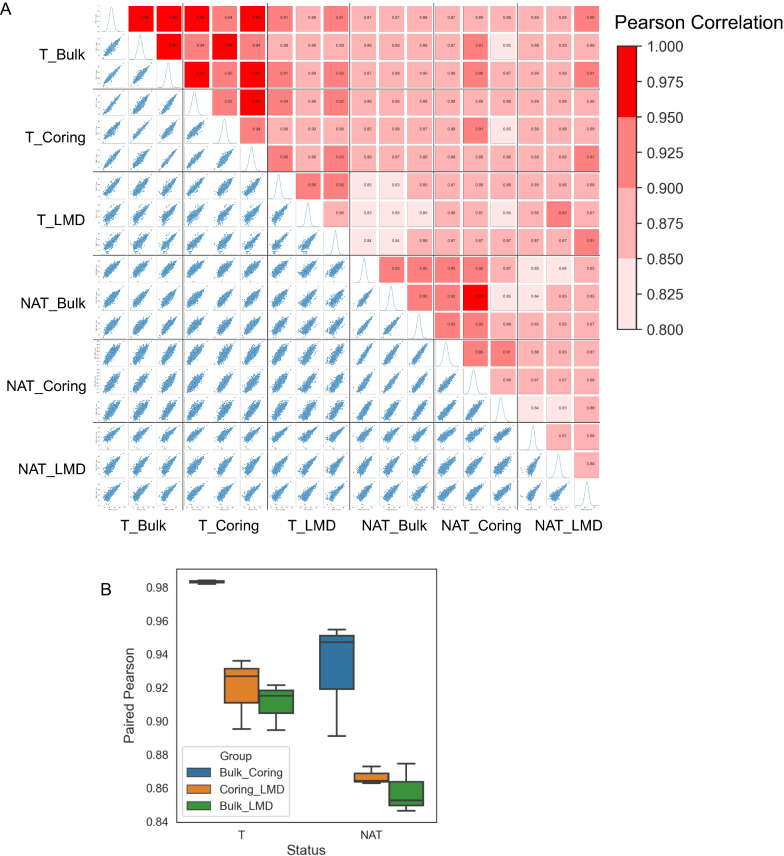
Correlation coefficient of quantified proteins in individual cases. **A** Pearson correlation coefficients of individual case in three types of samples and TMT sets. **B** Comparison of quantified proteins in bulk, coring and LMD samples

Proteomic analyses of samples from three sampling groups were characterized using TMT-labeling-based proteomics. In each tumor and/or NAT, over 8500 proteins were quantified (Additional file 1: Table S2). To evaluate the impact of three sampling techniques on proteomics, we compared protein abundances quantified from each tumor with NAT samples. Pearson correction coefficients of proteins from each tumor and NAT sample obtained from three sampling techniques ranged from 0.83 to 0.98 among six samples (three tumors and three NATs) (Fig. 2A). In the analysis of the same tissue obtained by different sampling techniques, the best correlation was found between bulk and cored samples, whereas the cored and LMD came as the second, and bulk and LMD samples showed the least correlation (Fig. 2B). In the pair-wise analysis of tumor or NAT tissues, tumor tissues showed higher correlation than NATs regardless which sampling technique was used to obtain the tissue sample (Fig. 2A, B).

Taken together, the LMD samples had lower correlation with bulk samples than did the cored samples, indicating LMD technique might have a stronger impact on the proteome composition compared to the coring technique.

### Protein expressional patterns in bulk, LMD, and coring samples

**Fig. 3:**
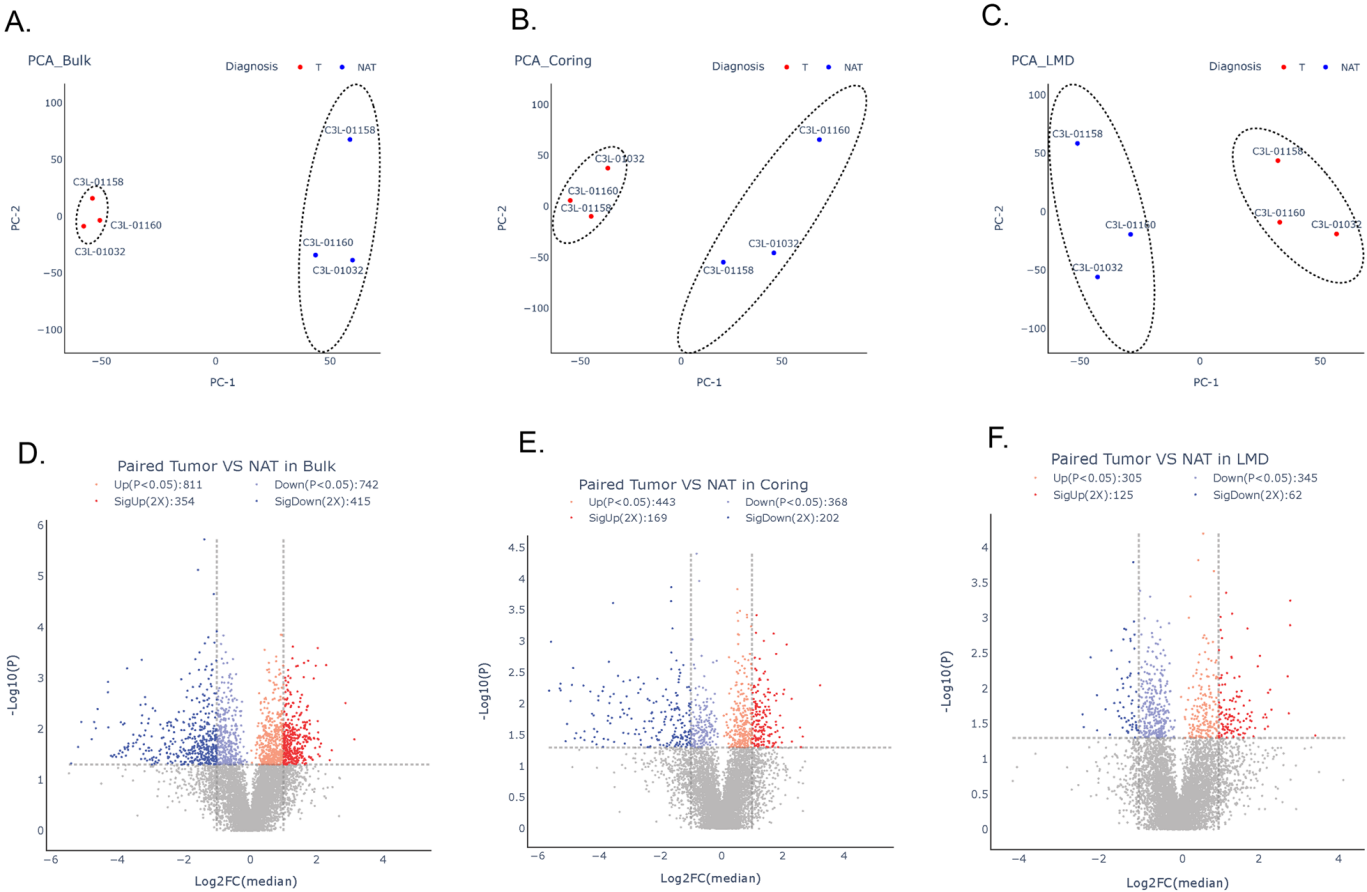
Principal component analysis and differential analysis of proteomic data from tumors and NATs from three sampling methods. **A** Analysis of tumors and NATs in bulk samples. **B** Analysis of tumors and NATs in coring samples. **C** Analysis of tumors and NATs in LMD samples, **D** Volcano plot of differentially expressed proteins in bulk samples, **E** Volcano plot of differentially expressed proteins in coring samples; **F** Volcano plot of differentially expressed proteins in LMD samples

Based upon the above quantified proteins, we investigated the protein expression patterns in the three sampling groups. PCA was performed and illustrated distinctive patterns between tumors and NATs (Fig. 3). All three sampling methods produced profiles that were separable from NATs (Fig. 3A–C).

A pair-wise comparison of the proteomic profile of tumor and NAT in the three sampling groups was conducted. In the analysis, the proteins considered differentially abundant and sampling-technique-associated proteins were those proteins with a log2 fold change ≥ 2 and adjusted *p*-value < 0.05. In the bulk tumors, 811 proteins were significantly up-regulated, and 742 proteins were significantly down-regulated in the tumors compared to NAT (Fig. 3D). In the cored tumor samples, 443 proteins were significantly up-regulated, and 368 proteins were significantly down-regulated in the tumors compared to NAT (Fig. 3E). In the LMD tumor samples, 305 proteins were significantly up-regulated, and 345 proteins were significantly down-regulated in the tumors compared to NAT (Fig. 3F).

**Fig. 4:**
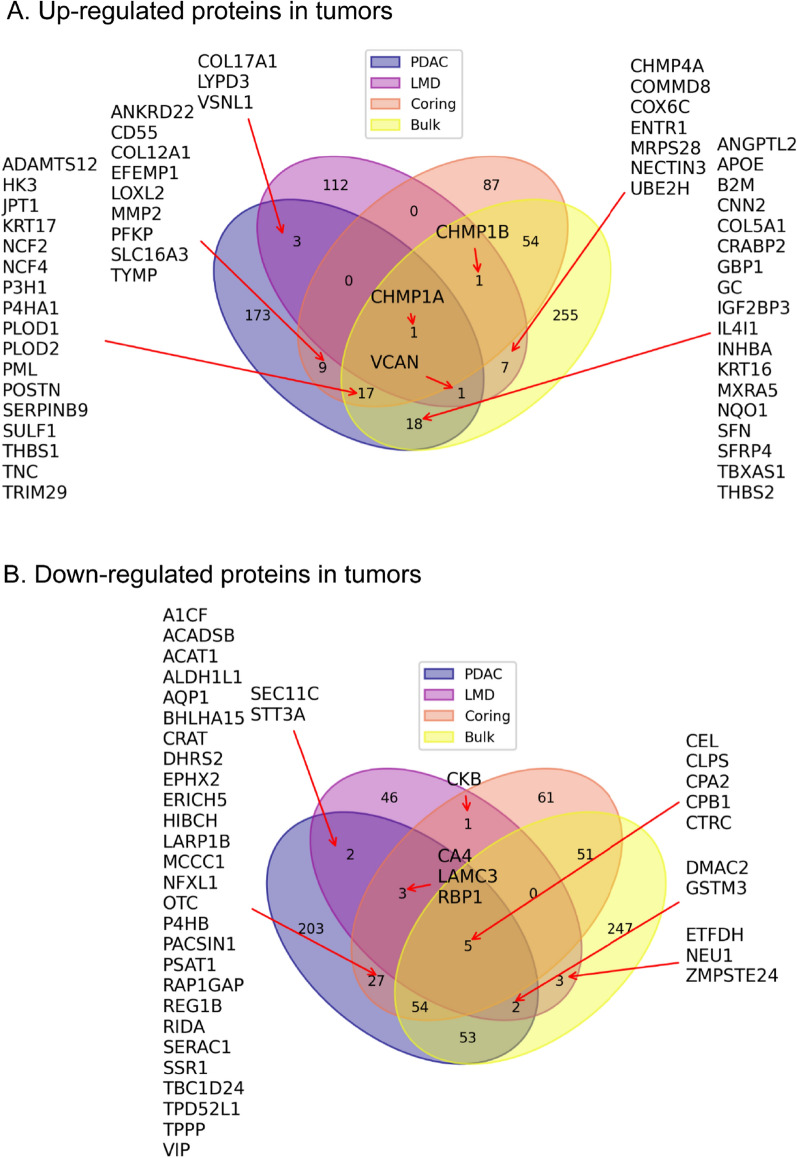
Identifications of changes of proteins in tumors sampled from three sampling methods. **A** Up-regulated proteins in tumors from the three sampling methods. **B** Down-regulated proteins in tumors from the three sampling methods

To evaluate the proteomic profile of tumor-associated proteins in different sample types, we compared significantly up- and down-regulated proteins in tumor samples with NATs (Fig. 4). Among up-regulated proteins, 115 and 96 tumor-associated proteins were uniquely identified in LMD and cored samples, which were not up-regulated in bulk samples. Only two proteins (CHMP1A and CHMP1B), members of the Endosomal Sorting Complex Required for Transport–III (ESCRT-III) family, showed a consistent elevation in all three sampling groups (Fig. 4A). Among down-regulated proteins, 52 and 92 tumor-associated proteins were uniquely identified in LMD and cored samples, which were not seen in bulk samples. Five proteins, including CEL, CLPS, CPA2, CPB1 and CTRC, showed a consistent down-regulated pattern in all three sampling groups (Fig. 4B).

To further evaluate the potential utility of cored and LMD for the enrichment of tumor cells, we compared proteomic signatures of bulk, cored, and LMD samples with a combined Pancreatic Cancer Database, including 2796 gene names of potential PDAC biomarkers [37, 38]. The database was used in our previous study [16]. The same two CHMP proteins were recorded in the Pancreatic Cancer Database (Fig. 4A). For tumor-associated proteins identified from the cored and bulk samples, 44 proteins were found in the Database, and 17 of them were commonly identified from bulk and coring techniques. Of tumor-elevated proteins identified in LMD sampling, 4 additional tumor-associated proteins, COL17A1, VSNL1, LYPD3, and VCAN, were reported in the Pancreatic Cancer Database (Fig. 4A).

In all, these data showed that distinct tumor-associated proteins were observed in samples obtained by different sampling techniques; However, findings might be limited by the small sample size and need to be further investigated in a large-scale study.

### Genomic-sequencing of KRAS mutations

Based upon the similarity of global proteomic data in bulk and cored samples and the distinct proteomic profiles in LMD samples (Fig. 2), we further evaluated the enrichment of neoplastic cellularity using the percent of variant allelic frequency (VAF) of *KRAS* mutations derived from WES as a surrogate signature for neoplastic cellularity for tumor tissues prepared by bulk, coring, or LMD. A total of eight PDAC tumors were included in the genomic analysis, including above three tumors and additional five PDACs. Of these additional five PDACs, the tumor cellularity ranged from 10 to 30% based on the pathology review of the digital histology images.

In the analysis, we determined and compared the VAFs of the *KRAS* mutations in tumor samples obtained by bulk, coring, and LMD techniques (Table 1). Two of three PDACs used in our proteomic analysis contained a relatively high neoplastic cellularity. In C3L-01032, *KRAS* percent of VAF were 34.2% in bulk and 48.1% in cored, and 45.9% in LMD samples, respectively, representing 68.4%, 96.2%, and 91.8% of neoplastic cellularity in the bulk, cored, and LMD samples, respectively. A similar pattern was observed in C3L-01160, with *KRAS* VAF scores of 29.5% in bulk 51.8% in cored, and 30.6% in LMD tumor tissues, respectively, representing 59.0%, 100.0%, and 61.2% neoplastic cellularity in the bulk, cored, and LMD samples. In the third PDAC used in proteomic analysis, C3L-01158, percent of *KRAS* VAF of 2.7% in bulk, 4.4% in cored, and 17.3% in LMD tumor tissues were observed, respectively, representing 5.4%, 8.8%, and 34.6% neoplastic cellularity in the bulk, cored, and LMD tumors (Table 1). In additional five tumor cases, *KRAS* VAFs ranged from 0.8% to 18.5% in bulk samples, 0.3% to 20.3% in cored samples, and 6.0% to 26.7% in LMD tumor samples were observed.

**Table 1:** The percent of variant allele frequencies (VAF%) of *KRAS* mutations in tumor tissues prepared by bulk, coring, and LMD techniques

Case	VAF (bulk)%	VAF (core)%	VAF (LMD)%	Fold changes (core/bulk)	Fold changes (LMD/bulk)	*KRAS* mutations
C3L-01032	34.2	48.1	45.9	1.4	1.3	p.G12D
C3L-01160	29.5	51.8	30.6	1.8	1.0	p.G12D
C3N-01897	18.5	20.3	26.7	1.1	1.4	p.G12R
C3N-02997	15.1	9.4	20.0	0.6	1.3	p.G12V
C3N-02591	10.3	9.1	21.2	0.9	2.1	p.G12R
C3N-02590	7.7	12.2	33.6	1.6	4.4	p.G12V
C3L-01158	2.7	4.4	17.3	1.6	6.4	p.G12R
C3L-03624	0.8	0.3	6.0	0.4	7.6	p.Q61H

The fold changes of VAFs of coring and LMD compared to VAF of the matched bulk tumors were included to show the neoplastic cellularity enrichment efficiencies by the coring and LMD methods

**Fig. 5:**
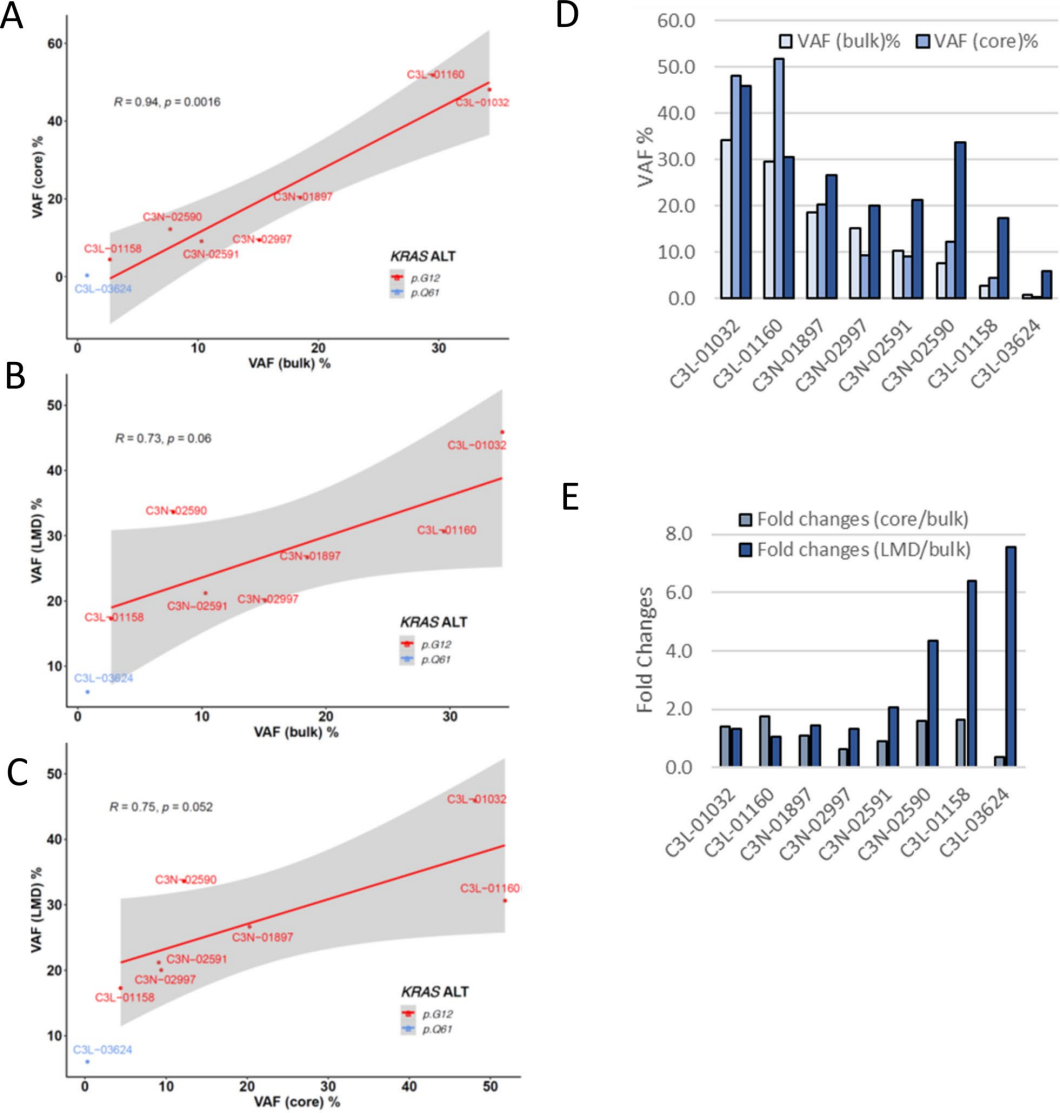
The variant allele frequencies (VAFs) of *KRAS* mutations in tumors prepared by bulk, coring, and LMD. **A** The scatterplot of percent of VAF for *KRAS* mutations based on the paired bulk and coring WES of 8 PDAC samples. **B** The scatterplot of VAFs of *KRAS* mutations based on the paired bulk and LMD of 8 PDAC samples. **C** The scatterplot of VAFs of *KRAS* mutations based on the paired cores and LMD of 8 PDAC samples. **D** The percent of VAF for *KRAS* mutations in 8 cases of PDAC tumors prepared by bulk, coring, and LMD methods. **E** The fold changes of VAFs from tumors prepared by coring or LMD comparing to the original VAFs from the bulk tumors

The scatterplot of *KRAS* VAFs between tumor samples prepared by bulk, coring, and LMD showed that cored tumor tissues were most similar to the bulk tumor (*R* = 0.94, Fig. 5A), while tumor tissues prepared by LMD showed less similarity to bulk (*R* = 0.73, Fig. 5B) or cored tissues (*R* = 0.75, Fig. 5C). These results are consistent with what we observed from the proteomic data, which showed a similar proteomic pattern between bulk and cored tissues while LMD showed distinct proteomic patterns (Fig. 2). The VAFs of bulk, cored, and LMD tumors showed that tumor tissues prepared by LMD method contained significantly higher neoplastic tumor cellularity ranging from 12.0% to 91.8% comparing to tumor tissues prepared by bulk or coring methods (Table 1, Fig. 5D). We further investigated the enrichment of neoplastic cellularity by coring and LMD methods using fold changes of *KRAS* mutations and found that LMD significantly enriched neoplastic cellularity. The enrichment of neoplastic cellularity by LMD is more effective for low cellularity bulk tumor tissues (Fig. 5E). The coring method showed some enrichment for neoplastic cellularity when the bulk tumors contained high neoplastic cellularity, but failed to enrich the neoplastic cells when the original bulk tumors contained low neoplastic cellularity (Fig. 5E).

## Discussion

The isolation of neoplastic cells plays a critical role in proteogenomic studies [12, 15, 16, 21]. As reported in the CPTAC PDAC proteogenomics and by others [16, 19, 21, 24, 39], PDAC tumor tissue is notorious for its highly variable tumor cellularity. The heterogeneous character of PDAC may obfuscate the study of tumor-associated proteins. The enrichment of neoplastic cellularity is the critical initial step in the proteogenomic profiling. Knowledge of the effect and differences of sampling techniques may allow us to refine proteomic profiles and, more importantly, to understand better the nature of PDAC for the early detection, monitoring the disease progression and response to therapy. The purpose of this study was to characterize PDAC tissues isolated from bulk, coring, and LMD using proteomics and genomics; and to evaluate the potential utility of coring and LMD in the enrichment of neoplastic cellularity. In our study, we included cases representing a spectrum of pathological features commonly seen in PDACs [39], with the neoplastic cellularity ranging from < 10% to > 80%. In addition, we also profiled pancreatic NAT components to include acinar and ductal cells.

In the global proteomic analysis, over 8500 proteins were identified in each sample regardless of sampling techniques. Tumor samples were distinguished from NATs by proteomic data obtained from all three sampling methods. These demonstrated that tumor-associated proteins can be determined using samples obtained by all three sampling techniques. The finding of comparative protein profiles of coring and bulk tissues demonstrated that cored and bulk tissues presented similar proteomic profiles. Our finding is critical for the analysis of clinical tumor samples, since the majority of PDAC patients present as advanced diseases, in whom a surgical resected bulk tumor sample cannot be obtained; as such, a tumor tissue can often be obtained by a biopsy procedure (similar to coring samples) for the characterization of the tumor.

To further evaluate tumor-associated proteins, we compared significantly up- and down-regulated proteins in each tumor sample using three sampling methods. Five pancreatic enzymes CEL, CLPS, CPA2, CPB1 and CTC, showed a consistent down-regulation in all three sampling methods. This makes sense as these proteins are strongly associated with non-neoplastic acinar cells. Among up-regulated proteins, only two proteins CHMP1A and CHMP1B showed a consistent elevation in all three sampling methods. These findings suggest that sampling methods used to enrich neoplastic cells can impact the proteomic findings.

Both CHMP1A and CHMP1B are members of the Endosomal Sorting Complex Required for Transport–III (ESCRT-III) family, and play critical role in transporting membrane-associated proteins such as receptor proteins into lysosome for degradation via the formation and sorting of multivesicular bodies [40]. In addition to their transporting/sorting function, a tumor suppression role of CHMP1A in kidney and pancreatic cancers has also been suggested [41, 42]. In an earlier study by Li et al., the tumor suppressor role of CHMP1A was indicated by growth inhibition of PanC-1 cells and conversion of non-tumorigenic human embryonic kidney cells to cells capable of forming xenograft tumors in athymic mice by stable knock-down of Chmp1A [41]. In the same study, authors also examined the expression of CHMP1A in human pancreatic tumors by immunochemical labeling of human pancreatic tumor TMAs, including 10 cases of pancreatic ductal carcinoma (PDACs). They found variable expressional patterns of CHMP1A in human pancreatic tumors, including a prominent expression of CHMP1A in PDACs. They also found that the CHMP1A protein showed a diffuse labeling pattern in PDAC cells compared with an apical labeling pattern in normal ductal epithelium. The IHC data demonstrated an obvious but mis-localized labeling pattern of CHMP1A in human PDAC [41]. In our study, we used fresh frozen PDAC tumor tissue and identified CHMP1A protein to be up-regulated in PDACs in the comparison with NAT. Our proteomic finding is similar to the IHC study by Li et al., that CHMP1A was detectable and elevated in human PDAC. Furthermore, studies have also demonstrated that CHMP1A is the only protein in ESCRT superfamily that contains a nuclear localization signal (NLS) at its N-terminus [43]. To further study the potential function of CHMP1A, Manohar et al. transformed PanC-1 cells with different truncated forms of Chmp1A, and found distinctive functional roles of the proteins [42]. The overexpression of NLS-deleted Chmp1A could promote the tumor cell growth, whereas, overexpression of C-terminal deleted Chmp1A could inhibit tumor growth [42]. Taken together, the tumor suppressor function of CHMP1A in cancers has been demonstrated in cell lines and animal models, also linked to the aberrant isoform of Chmp1a. However, the tumor suppressor role of CHMP1A in human cancer is still not well-studied, yet. Further evidence of its tumor suppressor role is needed.

To further understand the potential effect of coring and LMD on the enriched tumor-associated proteins, we compared tumor-associated proteomic signatures identified in bulk, coring, and LMD samples with the combined Pancreatic Cancer Database constructed from two studies [37, 38], including 2796 gene names of potential PDAC biomarkers as well as several highly robust biomarkers for the early detection and tumor progression. The database has been used in our recent proteogenomic study of 140 PDACs [16]. In current study, we found the two commonly identified proteins, CHMP1A and CHMP1B, were tumor-associated proteins in the Pancreatic Cancer Database. Of 115 tumor-elevated proteins identified in LMD sampling, 4 additional tumor-associated proteins, COL17A1, VSNL1, LYPD3, and VCAN, were reported in the Pancreatic Cancer Database. For tumor-associated proteins identified from the comparison of coring and bulk samples, 44 proteins were found in the Database, and 17 of them were to be commonly identified from bulk and coring techniques. These data demonstrated that abundances of tumor-associated proteins from cored and LMD methods might have distinctive enrichment patterns, suggestive of the impact of sampling techniques on the quantitative measurement of enriched tumor-associated proteins. However, our observation has several limitations, including a small number of study cases and suboptimal material in LMD samples.

The unique feature of LMD samples is suggestive of a LMD-specific proteome and warrants further investigations. Maurer et al. performed in-depth proteogenomic analysis of PDAC using LMD samples and compared the profile with that of bulk tissue [23]. In that study, they found unique signatures, and provided evidence of the differential expression and pathways in epithelial cell subtypes. Le Large et al., also identified the unique proteomic features of laser microdissected PDAC samples in comparison with bulk tissues [29]. They enriched tumor area and tumor-matched stroma using laser-capture microdissection and compared proteomic findings with the bulk tumor samples. They were able to identify 2 tyrosine kinase inhibitors targetable receptor tyrosine kinases (RTKs) in the tumor cell compartment obtained by laser-capture microdissection, including a previously known epidermal growth factor receptor 1 (EGFR) and a novel ephrin type-A receptor 2 (EPHA2). These proteins were not found in proteomes of stroma and bulk tumor samples, suggestive of tumor compartment-specific pathways. These studies indicate that laser-capture microdissection approaches can be used to enrich a desired area (compartment-specific areas) for highly selective proteomics [44]. However, the laser-based microdissection technique requires an extended tissue isolation procedure and prolonged separation time, which has a potential impact on the proteome and protein post-translational modifications (PTMs) in tumor-associated proteins and for subsequent multi-omics studies. The impact of LMD procedure requires further investigation. Nevertheless, LMD is an attractive approach for the LC-MS–based study of the composition of compartment-specific proteome within the tumor tissue.

To further evaluate the enrichment for neoplastic cells, we analyzed *KRAS* mutations in the tumor samples. *KRAS* alterations occur in > 90% of PDACs and mutations in *KRAS* therefore can be useful in estimating neoplastic purity [16, 19, 45]. We estimated the score of *KRAS* VAF as a surrogate of constituent tumor purity in samples. The increased scores of *KRAS* VAF in cored samples from C3L-0132 and C3L-01160 indicate that an enrichment of neoplastic purity near 100% can be achieved from bulk tumors containing high neoplastic cellularity by the coring technique, whereas, the *KRAS* VAF remained low in the other cored tumor samples from bulk tumors containing lower neoplastic cellularity. The genomic analysis of *KRAS* mutations revealed that tumor tissues prepared by LMD technique significantly enriched neoplastic cellularity, especially from bulk tumors with low neoplastic cellularity (Fig. 5E). Similarly to genomic findings, the significant impact of LMD on proteomic profile could due to the enrichment of neoplastic cellularity, however, the impacts of LMD technique to proteins could also play a role in the observed proteomic changes, which warrants further investigation. Taken together, these data suggest that LMD is most effective in enriching neoplastic cellularity from bulk tissues. Coring can enrich neoplastic cells from cancers containing a relatively high neoplastic cellularity. However, the coring technique seems to have a limitation for the isolation of neoplastic cells in cases with a low neoplastic cellularity. In our study, we only have small number of cases; a further study with a large cohort is necessary to further evaluate the utility of different sampling techniques.

Finally, our study is a preliminary work based on a small number of tumor cases. The aim of our study is not to identify tumor specific proteins or to select the ‘best performer’ from different sampling techniques, but rather to evaluate the impacts of different sampling techniques to proteomics and genomics and to provide insights and knowledge of each sampling technique. Although neoplastic cellularity is still an issue in the PDAC for proteogenomic analysis, especially in the study of tumor-associated proteins, single cell analysis techniques are now available to improve homogeneous cell inputs for quantitative proteogenomic measurements.

## Conclusions

In this study, we used different sampling techniques, including bulk sectioning of whole tumor tissue, LMD, and coring methods, for the proteogenomic analysis of PDAC. The protein signatures from cored samples revealed a comparative profile with bulk samples, whereas the protein profile from LMD samples demonstrated a unique signature, indicating the potential impact of the sampling techniques on the proteomic findings. In the genomic analysis, effective enrichment of neoplastic cellularity revealed by high *KRAS* VAF scores was found in LMD as well as cored tumor tissues obtained from certain bulk tumor tissues containing different amount of cellularity. Knowledge of the effects and differences of sampling techniques warrant further investigation.

## Supplementary Information

**Additional file 1: Table S1.** Clinical information of study cases. **Table S2.** Expression of proteins from the PDAC tumors and NATs

## Data Availability

All data generated and analyzed during this study are included in this published article and its supplementary information files. Pathology and radiology images can be accessed via Imaging Data Commons (IDC) at https://portal.imaging.datacommons.cancer.gov/explore/filters/?collection_id=cptac_ccrcc and The Cancer Imaging Archive at 10.7937/K9/TCIA.2018.OBLAMN27 [46].

## Abbreviations

ACN: Acetonitrile
AGC: Automatic gain control
bRPLC: Basic reversed-phase liquid chromatography
CHMP: Chromatin-modifying protein/charged multivesicular body protein
CPTAC: Clinical proteomic tumor analysis consortium
DDA: Data-dependent acquisition
DTT: Dithiothreitol; FA: formic acid
FDR: False discovery rate
H&E: Hematoxylin and eosin
HCD: Collision dissociation activation energy
HPA: Human Proteome Atlas
IAA: Iodoacetamide
iRT: Indexed retention time
LMD: Laser microdissection
MeOH: Methanol
MS: Mass spectrometry
NAT: Normal adjacent tissues
NCE: Normalized collision energy
PCA: Principal component analysis
PDAC: Pancreatic adenocarcinoma
PSM: Peptide spectrum match
QC: Quality control
RT: Room temperature
SD: Standard deviation
TCGA: The Cancer Genome Atlas
TCEP: Tris (2-carboxyethyl) phosphine
TFA: Trifluoroacetic acid
TMT: Tandem mass tag
VAF: Variant allele frequency
WES: Whole-exome sequencing
